# Unusual Presentation of Staphylococcal Scalded Skin Syndrome in an Elderly Patient With Acute Kidney Injury: A Case Report

**DOI:** 10.7759/cureus.56853

**Published:** 2024-03-24

**Authors:** Guntamukkala Geeta Sai, Sudhesshna Devi, Afthab Jameela Wahab

**Affiliations:** 1 Department of Dermatology, Saveetha Medical College and Hospital, Saveetha Institute of Medical and Technical Sciences, Saveetha University, Chennai, IND

**Keywords:** adult, skin biopsy, desquamation, acute kidney injury, staphylococcal scalded skin syndrome

## Abstract

Staphylococcal Scalded Skin Syndrome (SSSS) is characterized by denudation of the skin caused by *Staphylococcus *species. SSSS is common in infants, children, and rarely immunosuppressed adults or those with severe renal disease. We report a case of a 70-year-old female patient with an acute kidney injury who developed peeling of the skin over the axilla and back, which gradually spread to involve the upper and lower limbs, chest, and abdomen. A skin biopsy was performed, and a histopathological examination revealed a sub-corneal split consistent with SSSS. The patient was diagnosed with adult SSSS and was started on treatment with intravenous antibiotics, following which the skin lesions resolved.

## Introduction

Staphylococcal scalded skin syndrome (SSSS) represents a potentially life-threatening condition that presents with diffuse erythema and the formation of blisters over the whole body [1]. The large blistering areas of the skin give the appearance of a burn or scalding. Hence, the name staphylococcal scalded skin syndrome [2]. *Staphylococcus aureus* releases various toxins and enzymes, but only 5% of *Staphylococcus aureus* human isolates release exotoxins, exotoxin A (ETA) and exotoxin B (ETB), that cause SSSS [3,4]. These toxins target desmoglein-1, leading to a breakdown in the granular layer of the epidermis and the subsequent formation of bullae and denudation [3,5,6]. SSSS begins with a prodrome marked by fever, irritability, and fatigue [7]. The disease commonly starts as a local infection in the upper respiratory tract, ears, conjunctiva, umbilical stump, diaper area, circumcision, or other surgical wounds [1,8]. In adults, abscesses, arteriovenous fistula infections, septic arthritis, osteoarthritis, and pneumonia may cause this condition [1,9]. Within 24-48 hours, tender erythematous patches emerge typically starting on the face and flexural regions such as the neck, axillae, and groin. Several hours later, fragile blisters form within the erythematous patches that contain fluid ranging from thin, sterile liquid to frank pus [9]. These blisters then enlarge to form bullae that easily rupture, leading to skin peeling. The skin develops a characteristic wrinkled appearance due to the presence of the flaccid bullae, often referred to as "sad man facies” [10]. An intact blister on gentle pressure may extend laterally with a positive Nikolsky sign [8]. In the majority of cases, the skin heals without scarring within two weeks [1,8]. It is seen most commonly in infants and children, is rare in adults, and only over 50 adult cases have been documented until now [11].

We report a case of SSSS in a 70-year-old female with an acute kidney injury. She was treated successfully with systemic antibiotics.

## Case presentation

A 70-year-old female patient presented with a history of altered sensorium, urinary retention, breathlessness, and fever. Upon admission, laboratory investigations revealed elevated levels of urea and creatinine, a deranged complete blood count, and a deranged urine routine examination indicating acute kidney injury/urosepsis (Table 1).

**Table 1:** Laboratory values

Complete Blood Count	Reference range	Day 1	Day 3	Day 5	Day 10
Haemoglobin (g/dl)	Females: 12-15	10.6	8.4	8.2	10.1
Total red blood cell count (million/mm^3^)	Females: 3.8-4.8	4.10	3.23	3.08	3.74
Total leucocyte count (cells/mm^3^)	4000-10000	16670	13180	15360	11720
Absolute neutrophil count (cells/mm^3^)	2000-7000	12780	10110	11220	8200
Platelet count (lakhs/mm^3^)	1.5-4.5	3.20	3.70	3.87	4.49
Renal Function Test	Reference Range	Day 1	Day 3	Day 5	Day 10
Serum urea (mg/dl)	17-43	200	117	57	40
Serum creatinine (mg/dl)	0.7-1.4	2.2	1.2	0.8	0.9
Liver Function Test	Reference Range	Day 1	Day 3	Day 5	Day 10
Serum total bilirubin (mg/dl)	0.2-1.3	0.76	0.57	1.03	0.69
Serum direct bilirubin	0.1-0.4	0.37	0.22	0.67	0.33
Serum alkaline phosphatase (IU/L)	38-126	134	114	85	112
Serum albumin (gm/dl)	3.5-5.0	3.0	6.3	2.8	3.2

**Figure 1:**
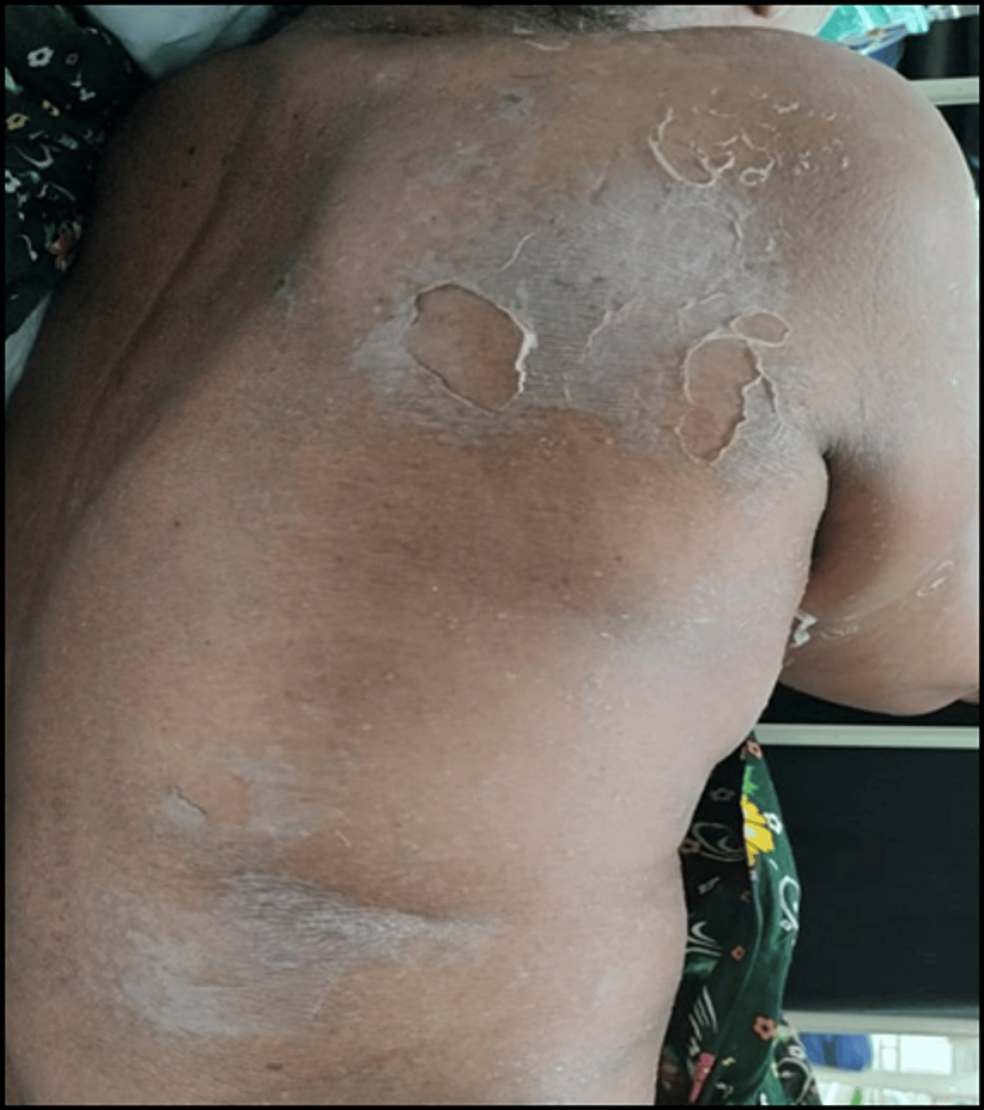
Multiple vesicles and exfoliation of skin over the back

**Figure 2:**
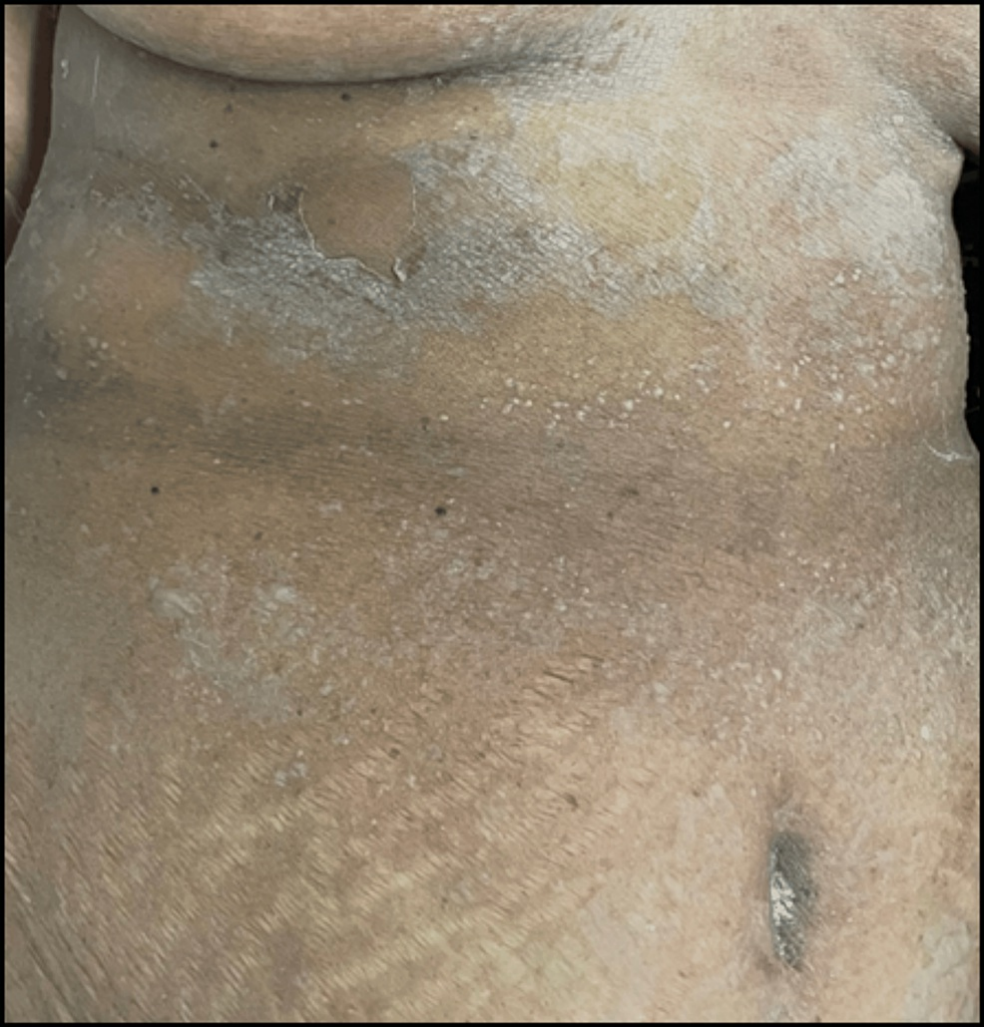
Multiple vesicles, bullae, and exfoliation of skin over the chest and abdomen

**Figure 3:**
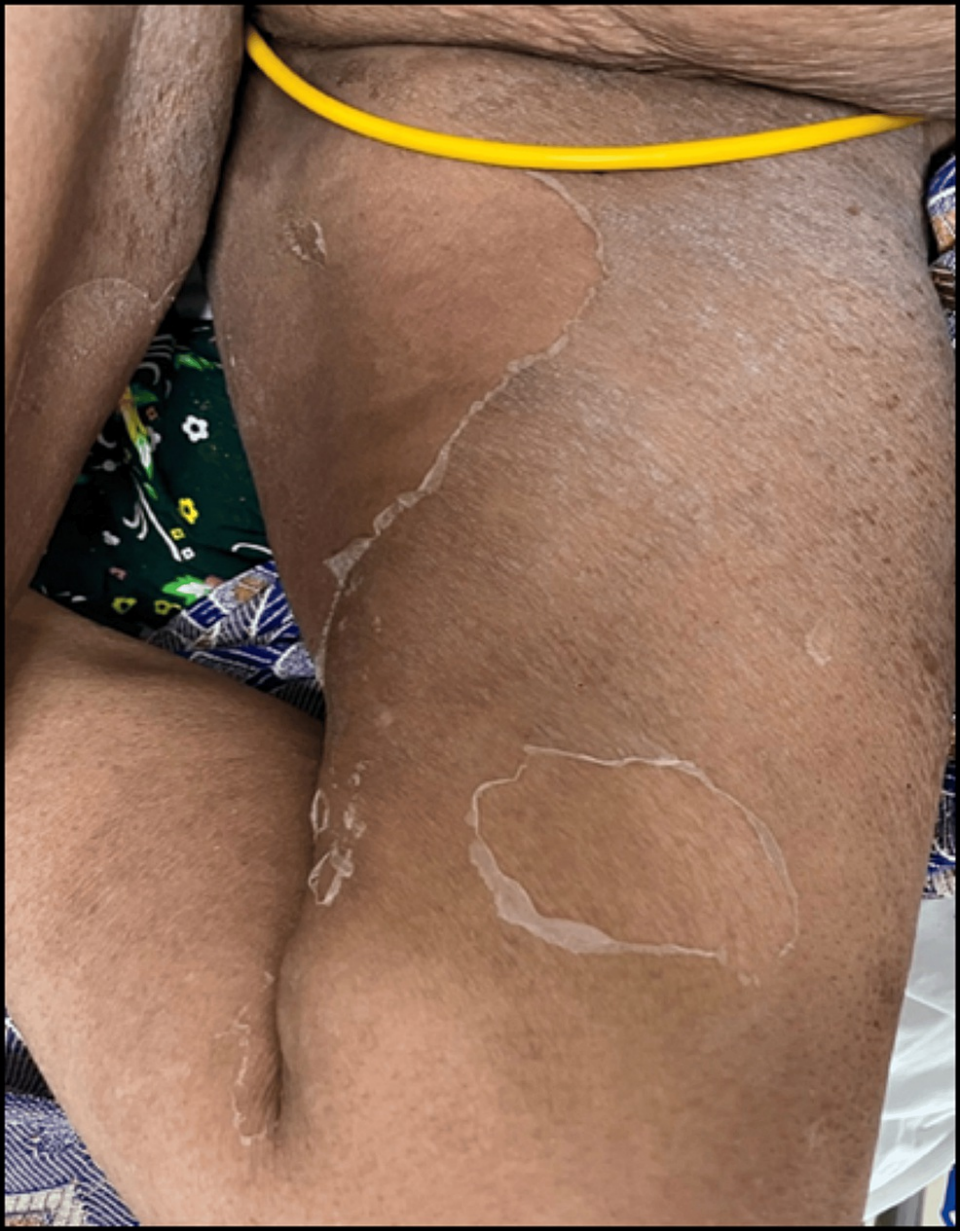
Exfoliation of skin over the thigh

The administered treatment included urinary catheterization, intravenous administration of antibiotics (cefoperazone + sulbactam following a test dose), IV fluid therapy, and Ranitidine injection alongside supportive measures. After five days of hospitalisation, the patient developed peeling of the skin over the axilla back that gradually spread to involve the upper and lower limbs, chest, and abdomen. A dermatology consultation was sought. On initial examination, desquamation was noted over the axilla, chest, abdomen, back, upper limbs, and lower limbs as shown in Figure 1, Figure 2, and Figure 3.

**Figure 4:**
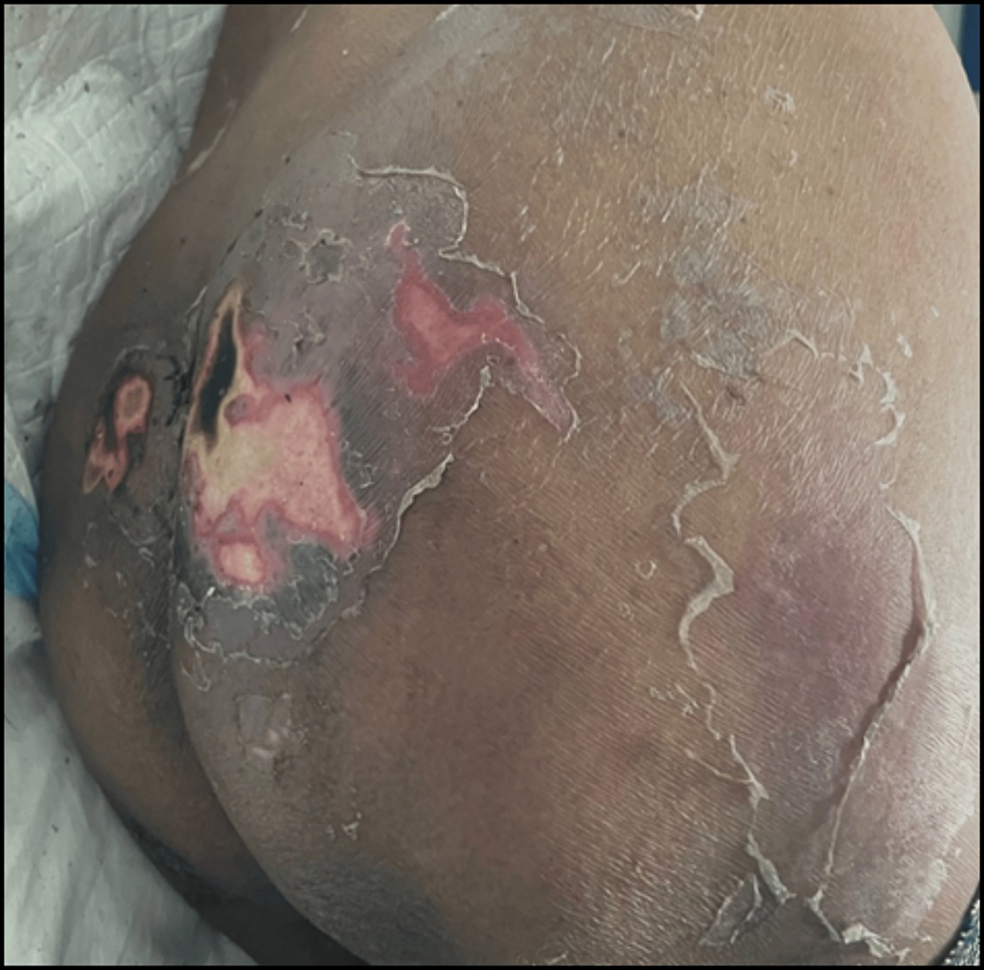
Erosive plaque with superficial sloughing and exfoliation of skin over the gluteal region

An erosive plaque with superficial sloughing was noted over the gluteal region (Figure 4). 

**Figure 5:**
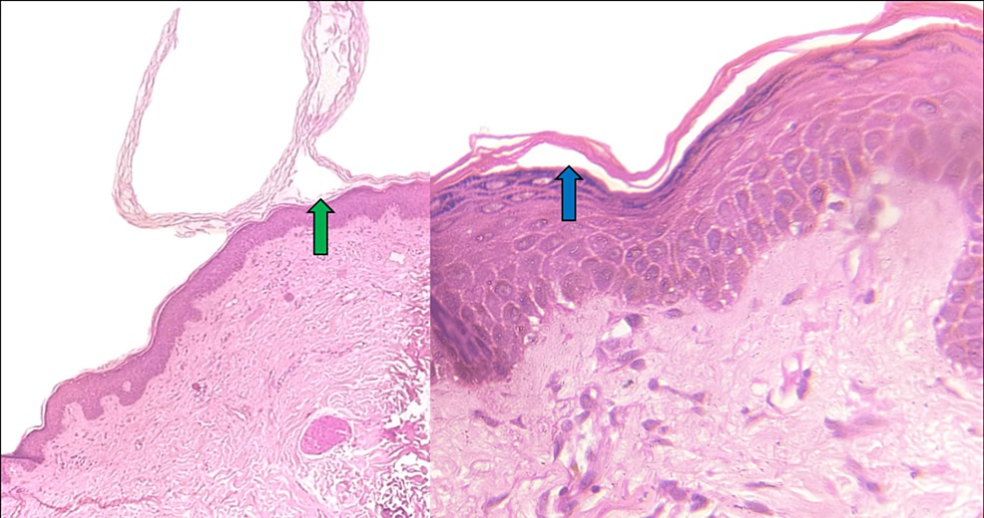
Skin histopathology revealing subcorneal split (haematoxylin and eosin) Green arrow: Scanning view; Blue arrow: Original magnification (x40)

The oral mucosa and genital mucosa were found to be normal. A skin biopsy was performed. Histopathological examination revealed a sub-corneal split consistent with SSSS (Figure 5).

The touch smear from the lesion was negative. The patient was diagnosed with adult SSSS. The blood culture sensitivity report from the femoral line indicated the presence of *Enterococcus *species, while the report from the central line showed Methicillin-resistant *Staphylococcus aureus* (MRSA). Both *Enterococcus *and MRSA demonstrated susceptibility to linezolid. The patient was then started on treatment with the antibiotic linezolid, following which the skin lesions resolved.

## Discussion

SSSS or Ritter disease is a potentially life-threatening disease caused by a *Staphylococcus aureus* infection. It is seen commonly in children below five years of age, affecting both sexes equally [1]. Adults seldom develop this condition because they possess antibodies against the exotoxin [9]. Two hypotheses for the increased occurrence of SSSS in children are either their lack of protective antibodies to counteract staphylococcal toxins or their kidneys are incapable of excreting the exfoliative toxin [1,12,13]. Adults who suffer from immunosuppression, renal failure, cancer, long-term alcohol misuse, or intravenous drug addiction are at risk for developing SSSS. Research revealed that reports of SSSS cases in healthy adults had also been made [11]. In adults, the mortality rate is as high as 60% [3,14,15]. However, in children, mortality is generally lower at <5%, and poor prognosis is associated with fluid and electrolyte imbalance sepsis [3,9,14].

Recent studies showed that about 46.8% of adults over the age of 70 have chronic kidney failure [16]. Ross and Shoff believed that in individuals with renal failure, the exotoxins are not eliminated, thereby contributing to the disease [9]. SSSS should be differentiated from bullous impetigo. Both result in blistering skin lesions due to staphylococcus exotoxin, but in bullous impetigo, the exotoxins are confined to the site of infection whereas, in SSSS, the exotoxins disseminate via the systemic circulation to distant areas to produce systemic symptoms. In this case, the diagnosis of SSSS was made after a histopathological examination revealed a sub-corneal split consistent with SSSS. It should also be differentiated from Steven Johnson syndrome (SJS), toxic epidermal necrolysis (TEN), acute generalized exanthematous pustulosis (AGEP), and drug reactions with eosinophilia and systemic symptoms (DRESS) (Table 2).

**Table 2:** Differential diagnosis SSSS: Staphylococcal scalded skin syndrome; SJS: Steven Johnson syndrome; TEN: toxic epidermal necrolysis; DRESS: Drug reaction with eosinophilia and systemic symptoms; AGEP: Acute generalized exanthematous pustulosis

Disease	Morphology	Histopathology	History of drug intake
SSSS	Bullae	Intraepidermal split	No
SJS/TEN	Bullae	Subepidermal split	Yes
DRESS	Morbilliform	Perivascular infiltrate with lymphocytes and eosinophils	Yes
AGEP	Erythema, pustules	Subcorneal pustules, apoptotic keratinocytes, spongiosis	Yes

Treatment includes supportive measures such as nasogastric feeding, intravenous fluids, intravenous antibiotics, and intravenous immunoglobulin (IVIG) therapy [17]. Urata et al. reported the successful treatment of adult SSSS patients with, in addition to antibiotics, IVIG therapy [18].

In the present case, the blood culture sensitivity report from the femoral line indicated the presence of *Enterococcus *species, while the report from the central line showed MRSA. Both *Enterococcus *and MRSA demonstrated susceptibility to linezolid leading to the initiation of linezolid therapy for the patient, following which the skin lesions resolved. Complications of SSSS are cellulitis, septicemia, scarring, and post-streptococcal glomerulonephritis. Early diagnosis with proper management in SSSS patients will have a good prognosis.

## Conclusions

This case report highlights the occurrence of SSSS in an adult patient with an acute kidney injury. Although SSSS is a rare condition, it should be considered a differential diagnosis in patients with extensive skin especially those with risk factors and immunosuppressive states. Early diagnosis and treatment with appropriate antibiotics result in a good prognosis. Knowing about the timely diagnosis and proper management of this condition is helpful for healthcare professionals.
